# Neutrophil-to-Lymphocyte Ratio Predicts Development of Immune-Related Adverse Events and Outcomes from Immune Checkpoint Blockade: A Case-Control Study

**DOI:** 10.3390/cancers13061308

**Published:** 2021-03-15

**Authors:** Pei Yi Lee, Kellynn Qi Xuan Oen, Grace Rui Si Lim, Juanda Leo Hartono, Mark Muthiah, Daniel Q. Huang, Felicia Su Wei Teo, Andrew Yunkai Li, Anselm Mak, Nisha Suyien Chandran, Chris Lixian Tan, Peiling Yang, E Shyong Tai, Kay Wei Ping Ng, Joy Vijayan, Yee Cheun Chan, Li Ling Tan, Martin Beng-Huat Lee, Horng Ruey Chua, Wei Zhen Hong, Eng Soo Yap, Dawn K. Lim, Yew Sen Yuen, Yiong Huak Chan, Folefac Aminkeng, Alvin Seng Cheong Wong, Yiqing Huang, Sen Hee Tay

**Affiliations:** 1 Department of Medicine, Yong Loo Lin School of Medicine, National University of Singapore, Singapore 119228, Singapore; peiyi.lee@mohh.com.sg (P.Y.L.); kellynn.oen@mohh.com.sg (K.Q.X.O.); e0057008@u.nus.edu (G.R.S.L.); leo_juanda@nuhs.edu.sg (J.L.H.); mark_muthiah@nuhs.edu.sg (M.M.); daniel_huang@nuhs.edu.sg (D.Q.H.); felicia_sw_teo@nuhs.edu.sg (F.S.W.T.); andrew_yunkai_li@nuhs.edu.sg (A.Y.L.); mdcam@nus.edu.sg (A.M.); nisha_suyien_chandran@nuhs.edu.sg (N.S.C.); chris_lixian_tan@nuhs.edu.sg (C.L.T.); Peiling_YANG@nuhs.edu.sg (P.Y.); mdctes@nus.edu.sg (ES.T.); Kay_WP_NG@nuhs.edu.sg (K.W.P.N.); joy_vijayan@nuhs.edu.sg (J.V.); Yee_Cheun_CHAN@nuhs.edu.sg (Y.C.C.); li_ling_tan@nuhs.edu.sg (L.L.T.); martin_lee@nuhs.edu.sg (M.B.-H.L.); Horng_Ruey_CHUA@nuhs.edu.sg (H.R.C.); wei_zhen_hong@nuhs.edu.sg (W.Z.H.); Eng_Soo_YAP@nuhs.edu.sg (E.S.Y.); mdcfa@nus.edu.sg (F.A.); 2 Division of Gastroenterology and Hepatology, Department of Medicine, National University Hospital, Singapore 119228, Singapore; 3 Division of Respiratory and Critical Care Medicine, Department of Medicine, National University Hospital, Singapore 119228, Singapore; 4 Division of Rheumatology, Department of Medicine, National University Hospital, Singapore 119228, Singapore; 5 Division of Dermatology, Department of Medicine, National University Hospital, Singapore 119228, Singapore; 6 Division of Endocrinology, Department of Medicine, National University Hospital, Singapore 119228, Singapore; 7 Division of Neurology, Department of Medicine, National University Hospital, Singapore 119228, Singapore; 8 Department of Cardiology, National University Heart Center, National University Hospital, Singapore 119228, Singapore; 9 Division of Nephrology, Department of Medicine, National University Hospital, Singapore 119228, Singapore; 10 Department of Laboratory Medicine, National University Hospital, Singapore 119228, Singapore; 11 Department of Ophthalmology, Yong Loo Lin School of Medicine, National University of Singapore, Singapore 119228, Singapore; dawn_lim@nuhs.edu.sg (D.K.L.); yew_sen_yuen@nuhs.edu.sg (Y.S.Y.); 12 Department of Ophthalmology, National University Hospital, Singapore 119228, Singapore; 13 Biostatistics Unit, Yong Loo Lin School of Medicine, National University of Singapore, Singapore 119228, Singapore; medcyh@nus.edu.sg; 14 Department of Haematology-Oncology, National University Cancer Institute, National University Hospital, Singapore 119228, Singapore; alvin_sc_wong@nuhs.edu.sg (A.S.C.W.); yiqing_huang@nuhs.edu.sg (Y.H.)

**Keywords:** neutrophil-to-lymphocyte ratio, immunotherapy, immune-related adverse events, survival

## Abstract

**Simple Summary:**

In this case-control study, we aimed to investigate the relationships between neutrophil-to-lymphocyte ratio (NLR) and platelet-to-lymphocyte ratio (PLR) with the occurrence of immune-related adverse events (irAEs) and clinical outcomes in cancer patients who had received at least one dose of immune checkpoint inhibitor. The study included 91 patients with irAEs and 56 controls. Multiple logistic regression showed that NLR < 3 at baseline was associated with higher occurrence of irAEs but PLR was not associated with development of irAEs. Multivariate Cox regression showed that development of irAEs and reduction in NLR from baseline to week 6 were associated with longer progression-free survival. Higher NLR values at baseline and/or week 6 were independently associated with shorter overall survival (OS). A reduction in NLR from baseline to week 6 was associated with longer OS.

**Abstract:**

The utility of neutrophil-to-lymphocyte ratio (NLR) and platelet-to-lymphocyte ratio (PLR) utility in predicting immune-related adverse events (irAEs) and survival have not been well studied in the context of treatment with immune checkpoint inhibitors (ICIs). We performed a case-control study of cancer patients who received at least one dose of ICI in a tertiary hospital. We examined NLR and PLR in irAE cases and controls. Logistic and Cox regression models were used to identify independent risk factors for irAEs, progression-free survival (PFS), and overall survival (OS). The study included 91 patients with irAEs and 56 controls. Multiple logistic regression showed that NLR < 3 at baseline was associated with higher occurrence of irAEs. Multivariate Cox regression showed that development of irAEs and reduction in NLR from baseline to week 6 were associated with longer PFS. Higher NLR values at baseline and/or week 6 were independently associated with shorter OS. A reduction in NLR from baseline to week 6 was associated with longer OS. In this study of cancer patients treated with ICIs, NLR has a bidirectional relationship with adverse outcomes. Lower NLR was associated with increased occurrence of irAEs while higher NLR values were associated with worse clinical outcomes.

## 1. Introduction

Immune checkpoint inhibitors (ICIs) have ushered in a new era in oncology and haematology, with these agents likely to become the backbone of cancer therapy in a wide range of cancer types [1]. However, the success, and therefore use of ICIs has also unveiled the unique challenge they pose—namely their association with immune-related adverse events (irAEs) and in certain cases, resulting in significant morbidity and mortality [2,3]. Interestingly, an association between irAEs with treatment response has also been described [4]. Given the significant role that irAEs play in cancer management and may affect long-term patient outcomes, studies have attempted to investigate the role of biomarkers in predicting the occurrence of irAEs. Notably, recent work has also demonstrated that elevated neutrophil-to-lymphocyte ratio (NLR) and platelet-to-lymphocyte ratio (PLR) were associated with poorer outcomes in several malignancies treated with ICIs [5,6,7]. However, the data on utility of these ratios in predicting irAEs is still emerging. In this case-control study, we aimed to investigate the relationships between NLR and PLR with the occurrence of irAEs and clinical outcomes in cancer patients who had received at least one dose of ICI. In addition, we reviewed the literature of published studies to investigate the association of these ratios with irAE occurrence and efficacy.

## 2. Materials and Methods

### 2.1. Patients

We performed a retrospective case-control study of patients aged 21 years or older treated with at least a single dose of anti-programmed cell death 1 (PD-1)/PD-1 ligand (PD-L1)/cytotoxic T-lymphocyte antigen 4 (CTLA-4)-based ICI at National University Hospital, Singapore from Jun 2014 to Sept 2019. Patients who had received combination ICI-based regimens were excluded. Controls were selected based on stage of cancer, type of cancer and class of ICI used. The study was approved by NHG Domain Specific Review Board B (reference code: 2017/01254) and was carried out in accordance with the principles of the Declaration of Helsinki. All subjects gave written informed consent prior to study inclusion.

### 2.2. Data Collection

Demographic data including age, gender, race, and clinical data such as Eastern Cooperative Oncology Group (ECOG) Performance Status (PS), body mass index (BMI), smoking status, and brain metastases were collected. Treatment data on the type, duration, and number of cycles of ICI, line of treatment and other cancer treatments, such as concomitant chemotherapy or radiotherapy during ICI were also collected. Patients received the following ICIs until tumor progression, development of unacceptable irAEs, withdrawal or death: nivolumab, pebrolizumab, atezolimumab, avelumab, durvalumab, and tremelimumab. irAEs were graded using the Common Terminology Criteria for Adverse Events v5.0. Patients suspected of having irAEs were reviewed through a chart review and only patients deemed to have irAEs were included as cases. Data was collected for patients with multiple episodes of irAEs, if any. These irAE episodes may be in the same or different organ systems. Progression-free survival (PFS) was calculated from the first day of treatment with ICIs to the date of radiographic or clinical progression or death, whichever came first. Overall survival (OS) was calculated from the first day of treatment with ICIs to the date of death from any cause. Blood counts data at baseline (most recent blood count before ICI initiation), at 6 (±2) weeks after therapy initiation, and at irAE diagnosis were used to calculate NLR (absolute neutrophil count/absolute lymphocyte count) and PLR (platelet count/absolute lymphocyte count). Patients with ongoing sepsis were excluded. All data were checked by the primary investigator (S.H.T.). Two other investigators (A.S.C.W and Y.H.) resolved any differences in the interpretation. We used PubMed to identify English studies that reported the association between NLR and PLR with irAE occurrence and efficacy of ICIs from database inception to August 2020. 

### 2.3. Statistical Analysis

The primary outcome was to evaluate the association of NLR and PLR with the development of irAEs in this case-control study. Secondary outcomes were to evaluate the prognostic values of NLR and PLR on clinical outcomes such as PFS and OS. NLR data were analyzed as a continuous variable or dichotomized into prespecified cutoffs for ≥3 versus <3 and ≥5 versus <5 [7]. PLR data were analyzed as a continuous variable or dichotomized into prespecified cutoffs for ≥180 versus <180 [7]. Normal distribution was assessed using the Shapiro-Wilk test. Continuous and categorical data were analyzed using the Mann-Whitney U test and Pearson’s chi-square test, respectively. The association between NLR and PLR and the development of irAEs was analyzed by univariate logistic regression. Variables that trended toward a significant association (*p* < 0.1) were further evaluated with multivariate analysis. When performing survival analyses, the dataset was considered as a whole. PFS and OS were calculated using the Kaplan-Meier method. Similarly, univariate followed by multivariate Cox regression models were employed to find independent predictors associated with PFS and OS. Statistical significance was defined as a two-tailed p value of <0.05. All statistical analyses were performed with SPSS, version 26 (IBM Corp, Armonk, NY, USA).

## 3. Results

### 3.1. Patient Characteristics 

Table 1 shows the comparison of characteristics between patients who experienced irAEs and those who did not. A total of 147 patients were included in the study, of which 91 (61.9%) were irAE cases and 56 (38.1%) were controls. The median age was 62.0 years (54.0–69.0) and the median time on follow-up was 10.2 months (3.7–19.6). The primary malignancies in this study were lung cancer (55.4%), colorectal cancer (7.4%), nasopharyngeal carcinoma (6.1%), gastric cancer (4.1%), hepatocellular carcinoma (4.1%), and others. The majority of patients had ECOG PS of 0-1 (87.2%). The irAE cases and controls had similar demographic, clinical and treatment histories. The median neutrophil, lymphocyte and platelet counts at baseline were similar in both groups. NLR and PLR at baseline and week 6 were not significantly different in both groups, although week 6 NLR trended higher in controls compared to irAE cases (*p* = 0.063). Endocrinopathies were the most common irAEs (8.1%), followed by hepatic (7.4%) and neurological (7.4%) disorders. Some patients developed multiple episodes of irAEs: 2 episodes in 32 (21.6%); 3 episodes in 15 (10.1%), and 4 episodes in 4 (2.7%).

### 3.2. Prognostic Role of NLR and PLR for the Development of irAEs

**Figure 1:**
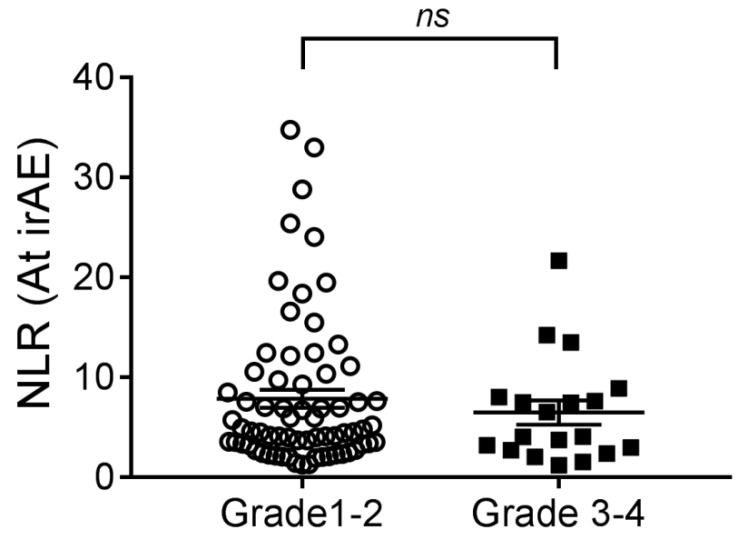
Comparison of neutrophil-to-lymphocyte ratio (NLR) between Grade 1–2 immune-related adverse event (irAE) and Grade 3–4 irAE at the first irAE episode. NLR was numerically lower in patients with higher grades of irAEs. The horizontal line represents the mean NLR and the standard error of the mean.

Univariate logistic regression was performed to assess the risk factors for irAEs (Table S1). Interestingly, patients with lung cancer, compared to other cancers, were less likely to develop irAEs (odds ratio (OR) = 0.49, 95% confidence interval (CI) 0.24–0.98, *p* = 0.044). Using the ratio data, baseline NLR < 3 (OR = 2.50, 95% CI 1.20-5.22, *p* = 0.015) was a risk factor for irAEs. Subsequent multivariate analysis revealed baseline NLR < 3 (adjusted OR = 2.27, 95% CI 1.07–4.82, *p* = 0.034) remained statistically significant in predicting development of irAEs (Table S2). However, the median NLR was not significantly different for Grade 1–2 irAEs versus Grade 3–4 irAE (4.57 vs. 4.04, *p* = 0.638) at the first irAE episode, though numerically lower in higher grades of irAEs (Figure 1). PLR was not associated with development of irAEs.

### 3.3. Univariate Analyses for PFS and OS

The median PFS and OS were 3.0 months (Q1:Q3 2.3; 3.7) and 10.6 months (Q1:Q3 7.5:13.8), respectively. According to the univariate analyses for PFS and OS, no significant differences were found with respect to age, gender, cancer stage, cancer type and class of ICI treatment. However, PFS and OS were reduced in patients with ECOG PS 2 but increased in patients on concomitant chemotherapy (Table S3). PFS was shorter in patients with higher baseline NLR, baseline PLR, week 6 NLR, week 6 NLR ≥ 3, and week 6 NLR ≥ 5 (Table S3). Reduction in PLR and NLR from baseline to week 6 was associated with longer PFS (Table S3). In the univariate analysis for OS, development of irAEs (HR 0.58, 95% CI 0.39-0.86, *p* = 0.006), higher BMI and reduction in NLR from baseline to week 6 were associated with longer OS (Table S3). OS was shorter in patients with higher baseline NLR, baseline NLR ≥ 3, baseline NLR ≥ 5, week 6 NLR, week 6 NLR ≥ 3, week 6 NLR ≥ 5, baseline PLR, baseline PLR ≥ 180, and week 6 PLR (Table S3).

### 3.4. Multivariate Analyses for PFS

Multivariable analyses demonstrated that development of irAEs (adjusted HR 0.67, 95% CI 0.45-0.99, *p* = 0.046) and reduction of NLR from baseline to week 6 (adjusted HR 0.56, 95% CI 0.33-0.95, *p* = 0.031) were independent prognostic factors for longer PFS (Table 2).

**Figure 2:**
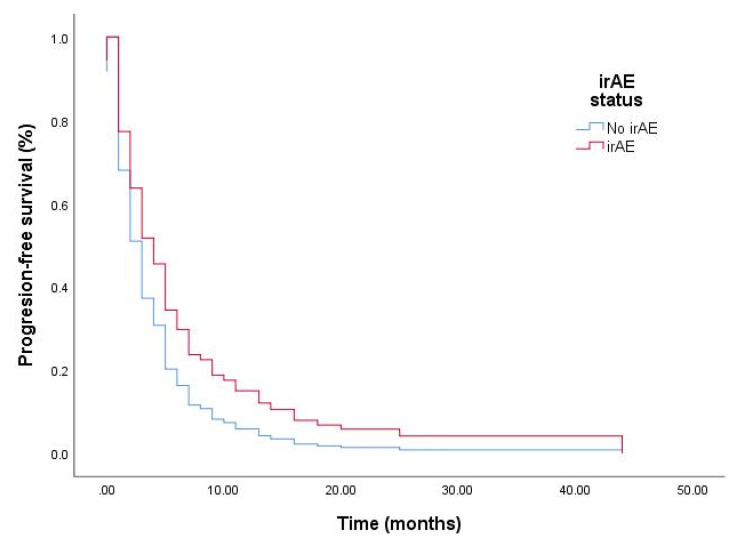
Adjusted survival curves for progression-free survival (PFS) according to irAE status. Comparison of survival curves in patients with or without irAE(s) for PFS.

Figure 2 illustrates the adjusted survival curves for PFS according to irAE status. Patients with irAEs had significantly longer PFS after ICI treatment compared to controls. Patients with reduction in NLR after ICI therapy had longer PFS (6.0 vs. 2.0 months, *p* < 0.001).

### 3.5. Multivariate Analyses for OS

In the multivariate Cox regression analyses, the NLR variables such as baseline NLR (adjusted HR 1.04, 95% CI 1.02-1.06, *p* = 0.001), baseline NLR ≥ 3 (adjusted HR 2.64, 95% CI 1.49-4.69, *p* = 0.001), week 6 NLR (adjusted HR 1.29, 95% CI 1.17-1.42, *p* < 0.001), week 6 NLR ≥ 3 (adjusted HR 3.12, 95% CI 1.73-5.61, *p* < 0.001), and week 6 NLR ≥ 5 (adjusted HR 3.55, 95% CI 1.84-6.85, *p* < 0.001) were independently associated with shorter OS (Table 3). Higher BMI and reduction of NLR from baseline to week 6 (adjusted HR 0.38, 95% CI 0.20-0.62, *p* < 0.001) were associated with longer OS (Table 3). Patients with reduction in NLR after ICI therapy had longer OS (18.2 vs. 6.4 months, *p* < 0.001). 

## 4. Discussion

Over the past decade, ICIs were introduced to the field of oncology and have since shown great clinical efficacy in reducing the disease burden in cancer patients [8]. As ICIs are increasingly employed in oncological treatment, it is important to look at the possible adverse events and overall outcomes associated with its use. NLR and PLR are simple and inexpensive biomarkers that can be obtained from basic laboratory tests commonly done in standard clinical practice. We focused on the NLR and PLR from the blood counts data as both biomarkers have been extensively studied in autoimmune diseases [9]. For example, the monocyte-to-lymphocyte ratio has not been described in association with systemic lupus erythematosus. In the present retrospective case-control study, we showed that baseline, week 6 and/or post-treatment reduction in NLR were effective in predicting complications (development of irAEs) and survival (PFS and OS). Our study also showed that patients who developed irAEs had longer PFS compared to those who did not. This finding is in keeping with a recent meta-analysis involving 4971 patients with multiple cancer types [10].

Neutrophil-to-lymphocyte ratio was first described by Zahorec et al. in 2001 and PLR was later described by Smith et al. in 2008 [11,12]. It is often asked if there is a biological relevance to these ratios. It is reasonable to speculate that the NLR is a measure of polymorphonuclear myeloid-derived suppressor cells (PMN-MDSCs) released from the bone marrow as a result of chronic inflammation [13]. The hallmark of PMN-MDSCs is their ability to suppress T lymphocyte function [14]. The most prominent factors implicated in MDSC suppressive activity include increased production of ROS and nitric oxide, upregulation of arginase I and production of prostaglandin E2 [15,16]. Importantly, Basu et al. reported a significant correlation between NLR and MDSCs recently [17]. We hypothesize that the biological activity of PMN-MDSCs suppressing T lymphocytes, expressed in the NLR, could shape the balance between autoimmunity and cancer. With regards to the PLR, the magnitude of stress-induced hypercortisolemia with subsequent release of platelets from the bone marrow and cortisol-induced lymphopenia result in the elevation of PLR in inflammatory disease states [18]. However, the PLR has not yet been ascribed to reflect the activity of any biological cell type.

Our study found that baseline NLR < 3 was a significant predictor for developing irAEs. Similar findings were noted in previously published cross-sectional studies, where reduced baseline NLR was found to be a significant risk factor for the development of irAEs (Table S4) [7,19,20,21]. Though the mechanism of irAEs is unclear, this finding is consistent with the hypothesis that NLR is a measure of PMN-MDSCs suppressing the non-specific inflammation and autoimmune response mediated by T lymphocytes due to the effect of ICIs [17,22]. Conversely, studies by Owen et al. and Peng et al. were not supportive of our findings (Table S4) [6,23]. Owen et al. noted no significant association between baseline NLR and irAEs, while Peng et al. noted that baseline NLR < 5 was associated with paradoxical lower risk of irAEs [6,23]. A majority of the patients in the study by Owen et al. were treated with nivolumab, and hence their results may not be fully applicable to patients treated with other ICIs. Another possible reason for this variation in findings could be attributed to the effects of genetics, age, gender, BMI, and other lifestyle or environmental factors on NLR and PLR, that may not have been fully taken into consideration in previous studies [24]. Association between low PLR and development of irAEs was not observed in our study. This is somewhat surprising, as it has been noted that the PLR is also an inflammatory marker in autoimmune conditions such as antineutrophil cytoplasmic antibody-associated vasculitis, Beçhet disease, rheumatoid arthritis, and systemic lupus erythematosus [18]. However, as discussed above, PLR may not be a surrogate marker of MDSCs like NLR and suppress autoreactive T lymphocyte function.

Elevated NLR and PLR have been shown to be significantly associated with worse outcomes in malignancies [25,26,27]. Specifically, similar findings have been reported in cancer patients who received ICI therapy (Table S5) [5,20,23,28,29,30]. In line with the literature, our study also noted that elevated NLR, and to a much lesser extent PLR, were associated with shorter OS and PFS in cancer patients who received at least one dose of ICI. However, as only 2 patients received anti-CTLA-4, the presented data should apply only to anti-PD1/PD-L1-based ICI. Biologically, the NLR can potentially reflect the balance of the immune system in the context of a malignancy. Controversy surrounds neutrophil function in cancer because neutrophils have been shown to possess both pro- and anti-tumor properties [31]. With tumor progression, dynamic changes in neutrophil composition result in a switch from an overall anti- to pro-tumor neutrophil contribution [31]. An increasing number of studies have linked increased peripheral blood neutrophils or NLR to tumor-infiltrating neutrophils. Gentles et al. analyzed cancer gene expression and clinical outcome data for approximately 18,000 patients and 39 distinct malignancies [32]. Of the tumor-associated leukocytes, neutrophil signature emerged as the most adverse cancer-wide prognostic population [32]. Lymphocytes are effective suppressors of cancer progression and their presence, particularly in the tumor microenvironment, is thought to reflect host immunity [33]. Another interesting finding in our analysis was the reduction in NLR, but not PLR, after ICI therapy was associated with significantly improved OS. Hence, NLR, unlike PLR, could also be a measure of PMN-MDSCs suppressing anti-tumor T lymphocyte responses in the tumor microenvironment. Of interest, anti-CTLA-4 treatment has been demonstrated to decrease PMN-MDSCs in patients with metastatic melanoma [34]. Therefore, the ability of NLR to reflect PMN-MDSC activity in anti-CTLA-4-based therapy needs to be examined.

The current study has several limitations and strengths. Our study is mainly limited by its retrospective nature and relatively small sample size. We did not analyze other inflammation-related peripheral blood markers such prognostic nutritional index and C-reactive protein-to-albumin ratio because not as many patients had data from liver function test and C-reactive protein [6,35]. In addition, our study population was obtained from a single center, which might limit generalizing the results to other countries or ethnicities. Future larger multi-center prospective studies would be useful in validating our findings. Lastly, although this is a case-control study involving multiple cancer types, the largest proportion of patients had lung cancer and this limits extrapolating the results to all cancer types as a whole. However, this study also has considerable strengths. This is the first study, to our knowledge, to employ a case-control study design to look at the association between NLR and the development of irAEs. First, as cases and controls were similar in terms of age, gender, and ethnicity, the effects of these variables as risk factors or confounders were not examined through use of logistic regression. Second, unlike other cross-sectional studies, the confounding effects of cancer stage and type on NLR and PLR were already accounted for during the study design stage. Third, our data was derived from multi-ethnic patients with different cancer types receiving various classes of ICIs, reflecting a real-world scenario. This makes demonstration of NLR to be an independent prognostic factor for irAEs, PFS, and OS in this heterogenous population all the more remarkable.

## 5. Conclusions

Our case-control study has demonstrated that NLR was independently associated with irAEs, PFS and OS. NLR has a bidirectional relationship with adverse outcomes. Lower NLR was associated with increased occurrence of irAEs while higher NLR values were associated with worse clinical outcomes. In addition, reduction of NLR at week 6 was associated with improved clinical outcomes. We propose that NLR is an inexpensive, readily available biomarker to predict different outcomes in cancer patients receiving ICI therapy. Prospective studies with large cohorts are warranted to validate the clinical application of our findings.

## Figures and Tables

**Table 1:** Comparison of characteristics of patients with or without immune-related adverse events (irAEs). Data are frequency (%) or median (interquartile range).

	Patients with irAEs (*n* = 91)	Patients without irAEs (*n* = 56)	*p*-Value
Age (years)	61.0 (52.0–70.0)	63.0 (55.5–68.0)	0.500
Gender (female)	31 (34.1)	17 (30.4)	0.641
Ethnicity			0.165
Chinese	67 (73.6)	44 (78.6)	
Malay	11 (12.1)	2 (3.6)	
Indian	2 (2.2)	4 (7.1)	
Others	11 (12.1)	6 (10.7)	
ECOG PS			0.949
0	30 (33.0)	19 (33.9)	
1	48 (52.7)	31 (55.4)	
2	2 (5.5)	4 (7.1)	
Smoking status			0.486
Smoker	40 (44.0)	23 (41.1)	
Non-smoker	35 (38.5)	26 (46.4)	
BMI	22.4 (19.2–25.0)	22.2 (20.6–23.5)	0.945
Cancer stage			0.380
I	6 (6.6)	1 (1.8)	
II	6 (6.6)	2 (3.6)	
III	13 (14.3)	9 (16.1)	
IV	54 (59.3)	41 (73.2)	
Cancer type			0.803
Lung cancer	44 (48.4)	37 (66.1)	
Renal cell carcinoma	1 (1.1)	1 (1.8)	
Nasopharyngeal carcinoma	6 (6.6)	3 (5.4)	
Melanoma	1 (1.1)	1 (1.8)	
Duration of ICI treatment (days)	72.5 (28.0–215.5)	68.0 (21.0–143.0)	0.225
No. of cycles	4 (2-11)	4 (2–8)	0.166
Class of ICI treatment			0.452
Anti-CTLA-4	2 (2.2)	0 (0.0)	
Anti-PD-1	54 (59.3)	37 (66.1)	
Anti-PD-L1	34 (37.4)	19 (33.9)	
Line of treatment			0.946
1st	25 (27.5)	15 (26.8)	
2nd	25 (27.5)	18 (32.1)	
3rd	18 (19.8)	10 (17.9)	
4th and beyond	21 (23.1)	12 (21.4)	
Concomitant chemotherapy	11 (12.1)	8 (14.3)	0.700
Concomitant radiotherapy	9 (9.9)	5 (8.9)	0.847
Brain metastases	9 (9.9)	8 (14.3)	0.432
Baseline FBC data (N × 10^9^/L)			
Neutrophil	4.29 (3.23–5.73)	5.49 (3.40–7.23)	0.122
Lymphocyte	1.30 (0.88–1.74)	1.25 (0.82–1.77)	0.603
Platelet	264 (195–342)	305 (213–367)	0.177
NLR			
Baseline	3.12 (2.22–5.93)	3.77 (2.92–7.49)	0.136
Week 6	3.20 (2.23–5.08)	4.21 (2.48–6.83)	0.063
PLR			
Baseline	211.84 (131.72–317.09)	213.00 (144.53–384.15)	0.582
Week 6	196.00 (134.97–337.79)	236.97 (172.62–383.85)	0.116
Organ system affected at 1st irAE			
Endocrine	12 (8.1)		
Hepatic	11 (7.4)		
Neurological	11 (7.4)		
Gastrointestinal	9 (6.1)		
Dermatological	8 (5.4)		
Pulmonary	5 (3.4)		
Rheumatic	3 (2.0)		

Abbreviations: ECOG PS, Eastern Cooperative Oncology Group Performance Status; BMI, body mass index; CTLA-4, cytotoxic T-lymphocyte antigen 4; PD-1, programmed cell death 1; PD-L1, PD-1 ligand; FBC, full blood count; NLR, neutrophil-to-lymphocyte ratio; PLR, platelet-to-lymphocyte ratio.

**Table 2:** Multivariate analyses of progression-free survival (PFS).

Variable	Category	PFS	
		Adjusted HR (95% CI)	*p* Value
Model 1			
ECOG PS	2	1.18 (0.49–2.83)	0.718
irAE status	irAE case	0.67 (0.45–0.99)	0.046
No. of cycles		0.91 (0.88–0.94)	<0.001
Concomitant chemotherapy		0.61 (0.33–1.13)	0.115
Baseline NLR		1.03 (0.98–1.09)	0.221
Baseline PLR		1.00 (1.00–1.00)	0.764
Model 2 *			
ECOG PS	2	1.10 (0.37–3.24)	0.862
irAE status	irAE case	0.77 (0.50–1.19)	0.240
No. of cycles		0.91 (0.88–0.95)	<0.001
Concomitant chemotherapy		0.83 (0.45–1.53)	0.546
Week 6 NLR		1.05 (1.00–1.11)	0.066
Reduction in PLR	Baseline/Week 6 PLR ratio ≥ 1	0.64 (0.40–1.04)	0.071
Model 3 *			
ECOG PS	2	1.10 (0.37–3.28)	0.865
irAE status	irAE case	0.76 (0.49–1.18)	0.226
No. of cycles		0.91 (0.88–0.94)	<0.001
Concomitant chemotherapy		0.80 (0.43–1.49)	0.485
Week 6 NLR	NLR ≥ 3	1.27 (0.80–2.00)	0.307
Reduction in PLR	Baseline/Week 6 PLR ratio ≥ 1	0.60 (0.37–0.95)	0.029
Model 4 *			
ECOG Performance	2	1.23 (0.42–3.61)	0.705
irAE status	irAE case	0.77 (0.49–1.18)	0.229
No. of cycles		0.91 (0.88–0.95)	<0.001
Concomitant chemotherapy		0.77 (0.42–1.42)	0.399
Week 6 NLR	NLR ≥ 5	1.17 (0.73–1.8)	0.521
Reduction in PLR	Baseline/Week 6 PLR ratio ≥ 1	0.59 (0.37–0.95)	0.031
Model 5*			
ECOG PS	2	1.37 (0.46–4.03)	0.574
irAE status	irAE case	0.72 (0.47–1.11)	0.141
No. of cycles		0.92 (0.88–0.95)	<0.001
Concomitant chemotherapy		0.93 (0.49–1.76)	0.811
Reduction in NLR	Baseline/Week 6 NLR ratio ≥ 1	0.56 (0.33–0.95)	0.031
Reduction in PLR	Baseline/Week 6 PLR ratio ≥ 1	0.73 (0.44–1.22)	0.228

* Patients on follow-up for at least 6 weeks included in the model. Abbreviations: PFS, progression-free survival; irAE, immune-related adverse event.

**Table 3:** Multivariate analyses of overall survival (OS).

Variable	Category	OS	
		Adjusted HR (95% CI)	*p* Value
Model 1			
ECOG PS	2	2.09 (0.82–5.36)	0.124
BMI		0.92 (0.86–0.97)	0.004
Cancer type	Lung cancer	0.78 (0.50–1.21)	0.268
irAE status	irAE case	0.68 (0.44–1.05)	0.084
No. of cycles		0.88 (0.84–0.93)	<0.001
Concomitant chemotherapy		0.52 (0.26–1.05)	0.066
Baseline NLR		1.04 (1.02–1.06)	0.001
Baseline PLR	PLR ≥ 180	1.12 (0.70–1.81)	0.631
Model 2			
ECOG PS	2	2.37 (0.96–5.87)	0.061
BMI		0.90 (0.85–0.95)	<0.001
Cancer type	Lung cancer	0.71 (0.46–1.10)	0.122
irAE status	irAE case	0.75 (0.48–1.16)	0.199
No. of cycles		0.86 (0.82–0.91)	<0.001
Concomitant chemotherapy		0.64 (0.32–1.26)	0.196
Baseline NLR	NLR ≥ 3	2.64 (1.49–4.69)	0.001
Baseline PLR	PLR ≥ 180	0.72 (0.41–1.27)	0.257
Model 3			
ECOG PS	2	2.48 (0.96–6.39)	0.060
BMI		0.91 (0.86–0.97)	0.003
Cancer type	Lung cancer	0.75 (0.49–1.17)	0.207
irAE status	irAE case	0.67 (0.43–1.04)	0.074
No. of cycles		0.88 (0.84–0.93)	<0.001
Concomitant chemotherapy		0.56 (0.28–1.12)	0.102
Baseline NLR	NLR ≥ 5	1.46 (0.87–2.43)	0.149
Baseline PLR	PLR ≥ 180	1.12 (0.68–1.84)	0.666
Model 4*			
ECOG PS	2	1.45 (0.46–4.61)	0.527
BMI		0.90 (0.84–0.97)	0.004
Cancer type	Lung cancer	0.94 (0.58–1.51)	0.791
irAE status	irAE case	0.72 (0.45–1.16)	0.178
No. of cycles		0.92 (0.88–0.95)	<0.001
Concomitant chemotherapy		1.18 (0.56–2.49)	0.663
Week 6 NLR		1.29 (1.17–1.42)	<0.001
Week 6 PLR		1.00 (1.00–1.00)	0.006
Model 5 *			
ECOG PS	2	2.23 (0.74–6.72)	0.156
BMI		0.91 (0.85–0.97)	0.006
Cancer type	Lung cancer	0.72 (0.44–1.18)	0.197
irAE status	irAE case	0.67 (0.42–1.08)	0.101
No. of cycles		0.89 (0.86–0.93)	<0.001
Concomitant chemotherapy		1.00 (0.47–2.12)	0.997
Week 6 NLR	NLR ≥ 3	3.12 (1.73–5.61)	<0.001
Week 6 PLR		1.00 (1.00–1.00)	0.823
Model 6*			
ECOG PS	2	2.81 (0.91–8.69)	0.074
BMI		0.93 (0.86–1.00)	0.035
Cancer type	Lung cancer	0.85 (0.53–1.38)	0.517
irAE status	irAE case	0.69 (0.43–1.10)	0.117
No. of cycles		0.91 (0.87–0.95)	<0.001
Concomitant chemotherapy		0.84 (0.41–1.72)	0.631
Week 6 NLR	NLR ≥ 5	3.55 (1.84–6.85)	<0.001
Week 6 PLR		1.00 (1.00–1.00)	0.395
Model 7*			
ECOG PS	2	4.06 (1.26–13.05)	0.019
BMI		0.94 (0.87–1.01)	0.071
Cancer type	Lung cancer	1.08 (0.68–1.73)	0.735
irAE status	irAE case	0.64 (0.40–1.02)	0.063
No. of cycles		0.90 (0.86–0.95)	<0.001
Concomitant chemotherapy		1.20 (0.56–2.59)	0.637
Reduction in NLR	Baseline/Week 6 NLR ratio ≥ 1	0.38 (0.20–0.62)	<0.001
Week 6 PLR		1.00 (1.00–1.00)	0.222

* Patients on follow-up for at least 6 weeks included in the model. Abbreviation: OS, overall survival.

## Data Availability

Data are not publicly available due to privacy and ethical restrictions.

## Supplementary Materials

The following are available online at https://www.mdpi.com/2072-6694/13/6/1308/s1, Table S1. Univariate binary logistic regression analysis to determine risk factors for irAEs; Table S2. Multivariate binary logistic regression analysis of factors associated with irAEs, Table S3. Univariate analyses of PFS and OS, Table S4. Studies on NLR and PLR in the development of irAEs, Table S5. Studies looking at the association of NLR and PLR with PFS and OS
