# Supplementary material for: Neutrophil-to-Lymphocyte Ratio Predicts Development of Immune-Related Adverse Events and Outcomes from Immune Checkpoint Blockade: A Case-Control Study
Source: Cancers (Basel). 2021 Mar 15;13(6):1308. doi: 10.3390/cancers13061308 (PMC8001500; doi:10.3390/cancers13061308)
Supplement: Supplementary file 1 [file cancers-13-01308-s001.pdf]

Table S1. Univariate binary logistic regression analysis to determine risk factors for irAEs.

Clinical variables	β (SE)	OR (95% CI)	p value
Age	-0.01 (0.01)	0.99 (0.96-1.02)	0.502
Gender			
Male	1 (Reference)	-	-
Female	0.17 (0.37)	1.19 (0.58-2.43)	0.642
ECOG PS			
0	1 (Reference)	-	-
1	-0.02 (0.37)	0.98 (0.47-2.04)	0.958
2	-0.23 (0.73)	0.79 (0.19-3.32)	0.750
Smoking status			
Smoker	0.26 (0.37)	1.29 (0.63-2.66)	0.487
Non-smoker	1 (Reference)	-	-
BMI	0.02 (0.05)	1.02 (0.93-1.11)	0.718
Cancer stage			
I	1 (Reference)	-	-
II	-0.69 (1.35)	0.50 (0.04-7.10)	0.609
III	-1.42 (1.16)	0.24 (0.03-2.36)	0.221
IV	-1.52 (1.10)	0.22 (0.03-1.90)	0.168
Cancer type			
Other cancers	1 (Reference)	-	-
Lung cancer	-0.71 (0.35)	0.49 (0.24-0.98)	0.044
Duration of ICI treatment	0.00 (0.00)	1.00 (1.00-1.00)	0.166
No. of cycles	0.02 (0.02)	1.02 (0.99-1.05)	0.308
Class of ICI treatment			
Anti-CTLA-4	1 (Reference)	-	-
Anti-PD-1	-20.83 (28419.64)	0.00	1.000
Anti-PD-L1	-20.62 (28419.64)	0.00	1.000
Line of treatment			
1st	1 (Reference)	-	-
2nd	-0.18 (0.45)	0.83 (0.35-2.01)	0.685
3rd	0.08 (0.51)	1.08 (0.40-2.95)	0.881
4th and beyond	0.05 (0.49)	1.05 (0.40-2.73)	0.920
Concomitant chemotherapy	-0.19 (0.50)	0.83 (0.31-2.20)	0.700
Concomitant radiotherapy	0.11 (0.59)	1.12 (0.36-3.53)	0.847
Brain metastases	-0.41 (0.52)	0.67 (0.24-1.84)	0.435
Baseline NLR			
NLR	-0.01 (0.02)	0.99 (0.95-1.03)	0.725
NLR < 3	0.92 (0.38)	2.50 (1.20-5.22)	0.015
NLR ⩾ 3	1 (Reference)	-	-
NLR < 5	0.40 (0.36)	1.50 (0.74-3.05)	0.266
NLR ⩾ 5	1 (Reference)	-	-
Baseline PLR			
PLR	0.00 (0.00)	1.00 (1.00-1.00)	0.914
PLR < 180	0.43 (0.36)	1.53 (0.76-3.09)	0.234
PLR ⩾ 180	1 (Reference)	-	-

Table S2. Multivariate binary logistic regression analysis of factors associated with irAEs. .

Independent variable	β (SE)	Adjusted OR (95% CI)	p value
Lung cancer	-0.57 (0.37)	0.57 (0.28-1.16)	0.119
Baseline NLR < 3	0.82 (0.39)	2.27 (1.07-4.82)	0.034

Table S3. Univariate analyses of PFS and OS.

Variable	Category	PFS		OS	
		**HR (95% CI)**	**p value**	**HR (95% CI)**	**p value**
Age		1.01 (1.00-1.03)	0.125	1.02 (1.00-1.03)	0.124
Gender	Female	1.03 (0.71-1.48)	0.884	1.02 (0.68-1.53)	0.924
ECOG PS	1	1.03 (0.70-1.50)	0.889	1.33 (0.86-2.04)	0.196
	2	2.83 (1.37-5.87)	0.005	6.79 (3.12-14.76)	< 0.001
Smoking status	Smoker	1.13 (0.78-1.66)	0.520	1.37 (0.90-2.09)	0.141
BMI		0.99 (0.95-1.04)	0.724	0.95 (0.90-1.00)	0.036
Cancer stage	II	0.58 (0.19-1.74)	0.330	1.07 (0.24-4.80)	0.927
	III	0.57 (0.23-1.44)	0.235	1.74 (0.51-5.96)	0.381
	IV	0.81 (0.35-1.87)	0.627	2.55 (0.80-8.12)	0.113
Cancer type	Lung cancer	1.29 (0.90-1.84)	0.163	0.68 (0.46-1.01)	0.056
irAE status	irAE case	0.71 (0.50-1.01)	0.057	0.58 (0.39-0.86)	0.006
Duration of ICI treatment		1.00 (1.00-1.00)	< 0.001	1.00 (0.99-1.00)	< 0.001
No. of cycles		0.92 (0.89-0.95)	< 0.001	0.88 (0.84-0.93)	< 0.001
Class of ICI treatment	Anti-PD-1	0.69 (0.17-2.82)	0.604	0.71 (0.17-2.90)	0.631
	Anti-PD-L1	0.55 (0.13-2.29)	0.412	0.52 (0.12-2.14)	0.361
Line of treatment	2nd	1.18 (0.74-1.87)	0.487	0.90 (0.53-1.52)	0.682
	3rd	1.06 (0.64-1.77)	0.827	0.96 (0.54-1.72)	0.893
	4th and beyond	1.01 (0.61-1.66)	0.974	1.10 (0.65-1.88)	0.722
Concomitant chemotherapy		0.54 (0.30-0.96)	0.035	0.48 (0.25-0.93)	0.029
Concomitant radiotherapy		0.86 (0.47-1.56)	0.618	0.89 (0.46-1.71)	0.731
Brain metastases		0.90 (0.52-1.57)	0.707	0.83 (0.45-1.56)	0.566
Baseline NLR	NLR	1.04 (1.02-1.06)	< 0.001	1.05 (1.03-1.07)	< 0.001
	NLR ⩾ 3	1.28 (0.90-1.84)	0.174	1.88 (1.25-2.83)	0.003
	NLR ⩾ 5	1.56 (1.08-2.25)	0.018	2.64 (1.77-3.96)	< 0.001
Week 6 NLR	NLR	1.11 (1.06-1.16)	< 0.001	1.24 (1.18-1.31)	< 0.001
	NLR ⩾ 3	1.67 (1.12-2.47)	0.011	2.91 (1.83-4.62)	< 0.001
	NLR ⩾ 5	2.15 (1.45-3.21)	< 0.001	3.85 (2.48-5.98)	< 0.001
Reduction in NLR	Baseline/Week 6 NLR ratio ≥ 1	0.48 (0.33-0.69)	< 0.001	0.37 (0.24-0.57)	< 0.001
Baseline PLR	PLR	1.00 (1.00-1.00)	0.024	1.00 (1.00-1.00)	0.001
	PLR ⩾ 180	0.97 (0.68-1.39)	0.884	1.52 (1.01-2.28)	0.044
Week 6 PLR	PLR	1.00 (1.00-1.00)	0.122	1.00 (1.00-1.00)	0.008
	PLR ⩾ 180	1.18 (0.79-1.75)	0.422	1.26 (0.81-1.96)	0.316
Reduction in PLR	Baseline/Week 6 PLR ratio ≥ 1	0.65 (0.45-0.93)	0.020	0.79 (0.52-1.17	0.238

Table S4. Studies on NLR and PLR in the development of irAEs.

Patients	N	Design	Indices	Results	Notes	Ref
**Supportive studies**						
Cancer patients who received at least one dose of pembrolizumab	391	Cross-sectional	dNLR	dNLR was significantly lower in patients with irAEs compared to the patients without irAEs (p = 0.005)<br><br>Univariate analysis: dNLR ⩾ 3 at baseline showed a negative correlation with the risk of developing irAEs (OR = 0.31, 95% CI 0.15-0.65, p = 0.002).<br><br>Multivariate analysis: Risk of irAEs was significantly lower in patients with dNLR ⩾ 3 at baseline (OR = 0.37, 95%CI 0.17-0.81, p = 0.012).	dNLR ⩾ 3 at baseline was associated with lower risk of developing irAEs.	Eun *et al.* [16]
Patients with melanoma who received either nivolumab or pembrolizumab	45	Cross-sectional	NLR	Univariate analysis: NLR at baseline (OR = 0.447, 95% CI 0.235-0.852, p = 0.0143) were significantly associated with development of vitiligo (skin irAE).<br><br>Multivariate analysis: No significant association found.	On univariate analysis, increased baseline NLR was associated with decreased risk of vitiligo.	Nakamura *et al.* [17]
Patients with metastatic NSCLC who received therapy with Nivolumab	92	Cross-sectional	NLR	Higher baseline NLRs were correlated with a lower frequency of irAEs [Δ: −0.36 (IQR −1.59–0.68) vs. 0.20 (IQR −0.86–2.32), p = 0.009].	Higher baseline NLRs were correlated with a lower frequency of irAEs.	Giannicola *et al.* [18]
Patients with advanced NSCLC treated with (cases) or without (controls) immune checkpoint inhibitors	184 cases, 79 controls	Cross-sectional	NLR, PLR	Univariate analysis: NLR < 3 and PLR < 180 at baseline were significantly associated with the development of irAEs (OR = 2.2, 95% CI 1.1-4.1, p = 0.018; OR = 2.8, 95% CI 1.4-5.5, p = 0.003 respectively).<br><br>Multivariate analysis: Only baseline PLR < 180 was confirmed as an independent predictive factor of irAEs (OR = 2.3, 95% CI 1.1-4.8, p = 0.027).	Baseline NLR < 3 and PLR < 180 were associated with higher risk of developing of irAEs.	Pavan *et al.* [7]
**Non-supportive studies**						
Patients with NSCLC treated with a single-agent immune checkpoint inhibitor	91	Cross-sectional	NLR, PLR	Baseline NLR ⩾ 5 and elevated baseline PLR > 237 were not associated with an increased risk of irAEs (p = 0.94; p = 0.45 respectively).	No association found between baseline NLR, PLR and irAEs.	Owen *et al.* [20]
Patients with histologically or cytologically proven diagnosis of advanced NSCLC (IIIB/IV), who were treated with anti-PD-1 antibodies	102	Cross-sectional	NLR	Univariate analysis: NLR < 5 at baseline was significantly associated with decreased risk of irAEs (OR = 0.051, 95%CI 0.02-0.14, p <0.001).<br><br>On multivariate analysis, NLR < 5 at baseline was an independent predictor of irAEs (OR = 0.04, 95% CI 0.01-0.13, p < 0.001).	NLR < 5 at baseline was associated with lower risk of developing irAEs.	Peng *et al.* [6]

Abbreviations: dNLR, derived neutrophil to lymphocyte ratio; NSCLC, non-small cell lung cancer.

Table S5. Studies looking at the association of NLR and PLR with PFS and OS.

Patients	N	Design	Indices	Results	Notes	Ref
Patients with metastatic non small cell lung cancer treated with Nivolumab	52	Cross-sectional	NLR, PLR	Univariate analysis: Elevated baseline NLR > 5 and PLR > 262 were associated with worse OS (HR for log(NLR) = 3.64, 95% CI 1.78-7.46, p < 0.001; HR for log (PLR) = 3.32, 95% CI 1.66-6.65, p = 0.001 respectively).<br><br>Multivariate analysis: Elevated baseline NLR > 5 was associated with worse OS and PFS (HR for log(NLR) = 5.01, 95% CI 2.03-12.37, p < 0.001; HR for log (NLR) = 2.09, 95% CI 1.22-3.58, p = 0.007 respectively).<br><br>No significant associations found between NLR and PLR with PFS on univariate analaysis.	Elevated NLR > 5 and PLR > 262 were associated with worse OS.	Diem *et al.* [5]
Patients with NSCLC who had undergone anti-PD-1 antibody treatment	54	Cross-sectional	NLR, PLR	Univariate analysis: Post treatment NLR ⩾ 5 at week 6 was associated with shorter OS and PFS (HR = 5.92, 95% CI 2.64-13.28, p < 0.001; HR = 23.75, 95% CI 7.56 - 74.66, p < 0.001 respectively). Post treatment PLR ⩾ 169 at week 6 was associated with shorter OS (HR = 1.56, 95% CI 0.78 - 3.12, p = 0.002).<br><br>Multivariate analysis: Post-treatment NLR ⩾ 5 at week 6 was independently associated with shorter OS and PFS (HR = 3.82, 95% CI 1.59-9.17, p = 0.003; HR = 15.09, 95% CI 4.55-50.06, p < 0.001 respectively).	Patients with elevated NLR ⩾ 5 at 6 weeks after treatment had worse OS and PFS.<br><br>Patients with elevated PLR ⩾ 169 at 6 weeks after treatment was associated with worse OS.	Suh *et al.* [26]
Patients with NSCLC treated with a single-agent immune checkpoint inhibitors	91	Cross-sectional	NLR, PLR	Patients with a baseline NLR ⩾ 5 had a shorter median OS compared to those with a lower NLR (p = 0.002).<br><br>No significant difference in median OS between patients with baseline PLR > 237 and those with lower PLR.	Patients with elevated baseline NLR ⩾ 5 had worse OS.	Owen *et al.* [20]
Patients with NSCLC who were treated with single-agent nivolumab after platinum failure	101	Cross-sectional	NLR	Patients with NLR ⩾ 3 at 2 and 4 weeks after nivolumab treatment were associated with shorter PFS (p = 0.00528; p = 0.00515 respectively).<br><br>Multivariate analysis: NLR at neither 2 or 4 weeks after treatment was associated with better PFS.	Patients with elevated NLR ⩾ 3 at 2 and 4 weeks after treatment had worse PFS.	Nakaya *et al.* [27]
Patients with advanced and refractory non small cell lung cancer	52	Cross-sectional	NLR	PFS was significantly shorter in patients with NLR ⩾ 5 compared to those with NLR < 5 (p = 0.014).	Elevated NLR ⩾ 5 was associated with worse OS and PFS.	Fukui *et al.* [28]

				OS was significantly worse in patients with NLR ⩾ 5 (p = 0.0003).<br><br>Univariate analysis: A pre-treatment NLR of ⩾ 5 was significantly associated with poorer OS compared to NLR < 5 (HR = 4.52, 95% CI 1.84-11.14, p = 0.001).<br><br>Multivariate analysis: A pretreatment NLR of ⩾ 5 was significantly associated with poorer OS compared to NLR < 5 (HR = 4.17; 95% CI 1.35-12.92; p = 0.013), independently.		
Patients with melanoma who received either nivolumab or pembrolizumab	45	Cross-sectional	NLR	On univariate and multivariate analysis, elevated baseline NLR was associated with shorter PFS (HR = 1.304, 95%CI 1.067-1.594, p = 0.0095).<br><br>Patients with baseline NLR >2.8 were associated with shorter PFS (HR = 3.094, p = 0.0032).	Elevated baseline NLR was associated with worse PFS.<br><br>NLR > 2.8 could be a useful baseline biomarker for indicating poor response to treatment.	Nakamura *et al.* [17]
Patients with metastatic non small cell lung carcinoma who received therapy with Nivolumab	92	Cross-sectional	NLR	Greater changes in NLR levels at third treatment course at 6 weeks vs. baseline was associated with worse PFS and OS (PFS: HR=1.19, 95% CI 1.06-1.34, p = 0.005; OS: HR = 1.27, 95% CI 1.10-1.46, p = 0.001).	Greater changes in NLR after treatment was associated with worse PFS and OS.	Giannicola *et al.* [18]
Patients with advanced NSCLC treated with (cases) or without (controls) immune checkpoint inhibitors	184 cases, 79 controls	Cross-sectional	NLR, PLR	Univariate analysis: Median PFS and OS were significantly shorter in patients with baseline NLR ⩾ 3 (HR = 1.797, 95% CI .220-2.645, p = 0.003; HR = 1.137, 95% CI 1.092-1.184, p < 0.001 respectively). Median PFS and OS were significantly shorter in patients with PLR ⩾180 (HR = 1.709, 95% CI 1.178-2.478, p = 0.005; HR = 1.004, 95% CI 1.002-1.005, p < 0.001 respectively).<br><br>Multivariate analysis: Median PFS and OS were significantly shorter in patients with baseline NLR ⩾3 (HR = 1.006, 95% CI 1.022-1.112, p = 0.003; HR = 1.098, 95% CI 1.032 - 1.169, p = 0.003 respectively). No significant association was found for elevated PLR.	Elevated NLR ⩾ 3 and PLR ⩾ 180 at baseline were associated with worse OS and PFS.	Pavan *et al.* [7]
Patients with a histologically or cytologically proven diagnosis of advanced NSCLC(IIIB/IV), who were treated with anti-PD-1 antibodies	102	Cross-sectional	NLR	Univariate analysis: NLR ⩾ 5 at baseline was associated with worse median OS and median PFS (HR = 2.311, 95% CI 1.375-3.882, p = 0.002; HR = 1.899, 95% CI 1.176-3.067, p = 0.009 respectively).<br><br>Multivariate analysis: NLR ⩾ 5 at baseline was associated with worse median OS and median PFS (HR = 2.491, 95% CI 1.288-4.819, p = 0.007;	NLR ⩾ 5 at baseline was associated with worse median OS and median PFS.	Peng *et al.* [6]

	HR = 1.845, 95%CI 1.002-3.399, p = 0.049 respectively).

Abbreviation: NSCLC, non-small cell lung cancer.
